# E-Cigarette Retailers’ Use of Instagram in New Zealand: A Content Analysis

**DOI:** 10.3390/ijerph20031897

**Published:** 2023-01-19

**Authors:** Lucy Hardie, Judith McCool, Becky Freeman

**Affiliations:** 1 School of Population Health, University of Auckland, Auckland 1142, New Zealand; 2 School of Public Health, The University of Sydney, Camperdown, NSW 2006, Australia

**Keywords:** smoking, social media, e-cigarettes

## Abstract

E-cigarette companies claim their products are key to improving health outcomes by providing smokers with lower-risk alternatives. However, the rapid uptake of e-cigarettes among young people has prompted concern about company marketing practices. In 2019, there was no legislation to govern e-cigarette marketing in New Zealand. This period provides an ideal context for examining how e-cigarette companies promoted their products before the introduction of marketing regulations. We conducted a content analysis of the Instagram accounts of five prominent e-cigarette retailers based in New Zealand during 2019–2020. We assessed health- and risk-related claims and marketing techniques. Less than 10% of Instagram posts refer to smoking alternatives or risk of nicotine addiction. E-cigarette devices were more likely to be promoted for stylistic features such as colours and ease of use (29.7%). Music festival sponsorship (19.1%), social media influencers (9.2%), and lifestyle marketing (41.5%) were identified as youth-oriented promotional strategies. E-cigarette retailers claim to promote harm-reduction tools to smokers, yet this study finds few references to smoking alternatives in any content. Instead, retailers utilised strategies to engage with a young audience, including festival sponsorship and stylish influencers. This youth-oriented marketing, in combination with weak and delayed government action, may have contributed to the high use of e-cigarettes among young New Zealanders.

## 1. Introduction

E-cigarette manufacturers have positioned their products as key to reducing smoking related illness [1]. There is some evidence that e-cigarettes may help smokers quit in a clinical setting compared to traditional nicotine replacement therapy [2]. Nevertheless, studies at the population level indicate that the use of e-cigarettes is increasing beyond smokers and is now one of the main sources of substance use among young people and non-smokers. Among New Zealand youth (15–24 years), daily e-cigarette use has increased substantially from 3% to 18.6% between 2017 and 2022 [3]. The marketing strategies used by retailers to promote highly addictive products have come under scrutiny as youth uptake has increased [4,5]. There is near universal agreement that young people should be protected from tobacco-related marketing. Yet, regulatory policies in many countries have not kept pace with current tobacco and vaping industry marketing strategies in retail settings and on digital platforms [4,5,6].

Social media has played a central role in the marketing and popularisation of e-cigarettes among young people globally [7,8,9]. Exposure to e-cigarette content on social media has been associated with positive attitudes towards, subsequent use, and lower perceived risk of harm of e-cigarettes [8,10]. Brands have effectively used social media to promote e-cigarettes as on-trend devices using stylistic techniques described as “patently youth-oriented” [11], positioning products within youth social groups, style, and activities [12]. Examples of youth-oriented e-cigarette marketing include the promotion of candy flavours, vape cloud tricks, brightly coloured devices, and the involvement of young influencers [13,14].

Instagram is a social media platform with over one billion users worldwide [15]. In 2019, Instagram use in New Zealand surged compared with other social media platforms, such as Facebook, and was particularly popular with young people [16]. The immediate visual appeal of Instagram, which is primarily image-based, is considered the core driver behind the platform’s popularity [17]. Image-based social media platforms enable brands to visually position their products according to the ideals, aspirations, and style of the desired target audience, known as ‘lifestyle marketing’ [18]. This strategy aims to enhance social acceptance of products by portraying notions of friendship, freedom, and enjoyment; ideals and imagery particularly appealing to young people [19].

Historically, tobacco companies have used lifestyle marketing strategies to create positive associations with their brands. Modern e-cigarette marketing practices and promotions have similarities with this model [13,20,21]. For example, brands have encouraged social acceptance by promoting e-cigarettes at social events, such as parties and music festivals [22]. Social media capabilities enable rapid and highly targeted creation and dissemination of content.

Before 2018, New Zealand regarded e-cigarettes as oral tobacco products and prohibited their sale under its legislation [23]. However, following a court case, the Ministry of Health v Philip Morris International, the judge ruled that New Zealand’s tobacco control legislation did not legally cover aerosol products [24]. As a result of this ruling, retailers were able to promote and sell e-cigarettes immediately and freely. The government enacted e-cigarette-specific legal restrictions in November 2020, meaning companies promoted their products without restriction for more than two years. For example, billboards, television, and radio advertisements were commonplace [25] and even the sale of products to people under 18 was not legally prohibited. Almost all New Zealand e-cigarette retailers are active on social media [26,27]. The period prior to the adoption of New Zealand legislation to regulate e-cigarettes provides an important context for examining how e-cigarettes are marketed on social media in the absence of any government regulation.

This study aims to examine the Instagram content of prominent e-cigarette retailers in New Zealand and to analyse the marketing techniques and health claims in the period prior to the adoption of e-cigarette marketing legislation.

## 2. Materials and Methods

A content analysis of Instagram posts was conducted to determine the health claims and marketing strategies New Zealand e-cigarette retailers employed.

### 2.1. Sample

In August–September 2019, we identified 36 prominent e-cigarette retailers in New Zealand using the internet search engine Google using variations of search terms ‘e-cigarette’ AND ‘buy’ AND ‘New Zealand’ as described in full in Hardie et al., 2021 [27]. Retailers were then ranked by the number of Instagram followers. The five retailers with the largest number of followers were included in the sample: Vapo, Shosha, Cosmic, NZVapor, and Vype (rebranded in 2020 as Vuse, owned by British American Tobacco [BAT]). Instagram posts were collected retrospectively in 2022 for the period April 2019 to March 2020. Where a singular post contained multiple images, each image was coded individually. Instagram Stories are ephemeral posts which disappear after a period of 24 h. These were collected during the period 29 December 2019 to 29 January 2020 to capture temporary content. Posts were screen-captured and extracted into Excel v 16.60. Images included in this paper as examples have had personal identifiers redacted. One retailer promoted products other than e-cigarettes (e.g., candles and soaps), these posts were excluded.

### 2.2. Coding Framework

We created a coding framework based on previous research on unhealthy commodity promotion on social media [14,28]. Modifications to the coding frame were made during iterative testing to ensure it met the study aims of capturing relevant health claims and marketing themes. All three authors discussed the coding structure, and decisions to amend, remove, or add categories were made by consensus. Once the codebook was finalised, all authors coded a random sample of 30 posts. A high level of agreement was achieved (94.6%), and the first author (LH) coded the remaining posts in the sample. The coding framework and definitions are outlined in Supplementary Table S1.

### 2.3. Coding Process

Using a checklist procedure, we recorded whether each item was present (1) or absent (0). We included the image or video and any descriptive text in the caption in our assessment.

Our research focused on images and information presented by e-cigarette retailers regarding (1) lifestyle marketing, including associations with events, and the use of social media influencers, (2) claims about health risks or benefits, and (3) marketing techniques such as competitions and giveaways, price promotions, and product type as defined in Supplementary Table S1. Images containing more than one aspect of interest, such as a pod device and a giveaway, were both coded to gather as much data as possible. To measure consumer engagement, we recorded the number of ‘likes’ each post received for image-based posts or the number of views for video posts. For competitions in which entries were made by tagging friends, we exported the tags into Microsoft Excel to determine the number of entries. Instagram influencers are generally defined as individuals tagged in a post or Story with more than 1000 followers [29]. However, recent evidence indicates that influencers with small followings can be highly influential as relationships are perceived to be stronger [30]. To reflect this knowledge and the small New Zealand context, we included influencers with >500 followers.

## 3. Results

A total of 513 Instagram posts were obtained for the time period. Sixty-two of these were Stories (24 h posts). Total posts by retailer are outlined in Table 1. Retailer Vapo accounted for the largest number of posts, followed by Vype. Vapo also had the largest number of followers.

### 3.1. Health Related Statements

**Figure 1:**
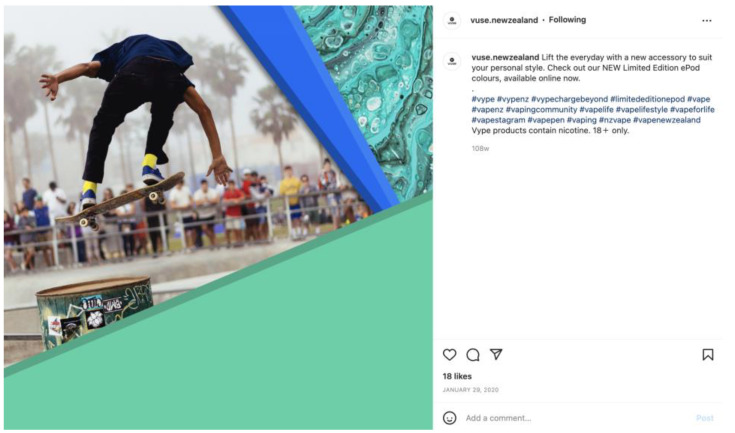
Example of an Instagram post that states the product contains nicotine.

Less than 10% (*n* = 50) of the total Instagram posts referred to e-cigarettes as safer smoking alternatives (Table 2). Thirty-two percent (*n* = 144) of posts stated that products contain nicotine. The majority of these statements (86% *n* = 127) were made by one company (Vype). Statements were written in small font following the text captions and hashtags (Figure 1). References to the risk of addiction were present in just 7.6% of posts (*n* = 39). Table 2 shows descriptive statistics of post characteristics as evaluated using the checklist procedure.

### 3.2. Device Features

**Figure 2:**
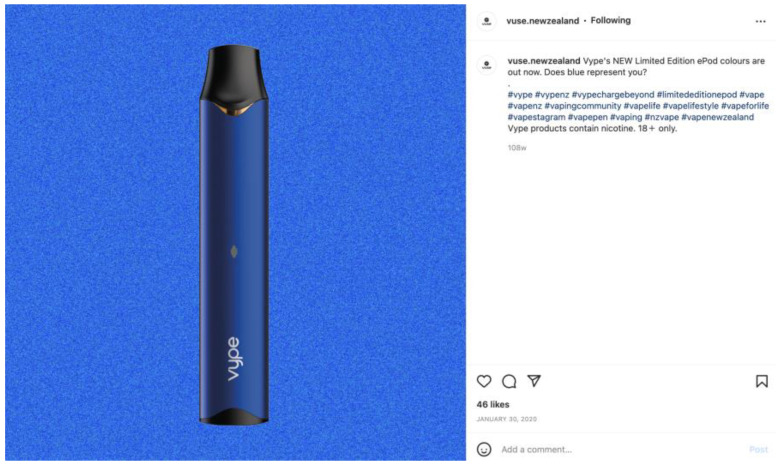
Example of an Instagram post promoting a personalised style feature.

Of the 210 Instagram posts that showed an image of an e-cigarette device, most (*n* = 157) were pod devices. No posts promoting disposable devices were identified. E-cigarette devices were most likely to be promoted for style features such as a range of colours or discrete design (Figure 2).

### 3.3. Events

**Figure 3:**
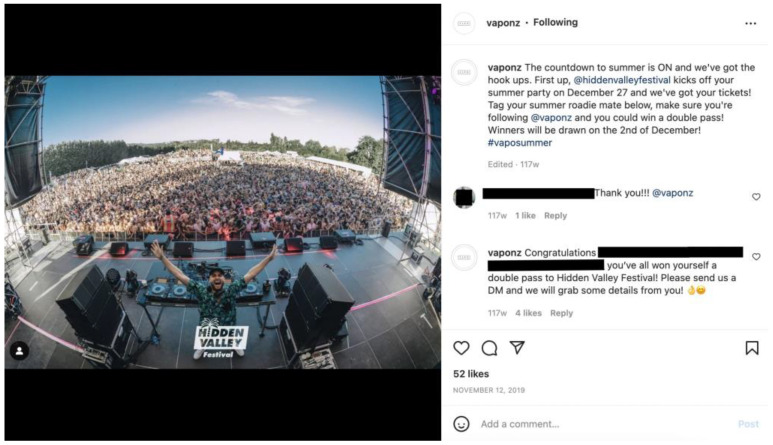
Example of an Instagram post promoting a music event.

Twenty-three per cent (*n* = 117) of all posts in the sample related to the promotion of sponsored events (Figure 3). The majority of those were music festivals and concerts (*n* = 98) including prominent summer festivals Rhythm & Vines and Sound Splash. Brands also sponsored and promoted motorsports (*n* = 9), expos (*n* = 6), and other events, such as boxing matches (*n* = 4).

### 3.4. Giveaways and Competitions

**Figure 4:**
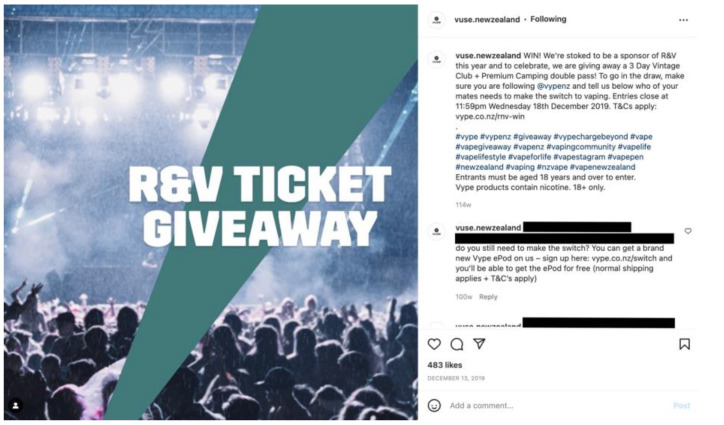
Example of an Instagram post promoting a music festival giveaway.

Nearly 20% (19.7%) of posts included giveaways or competitions in which winners were rewarded with products (*n* = 46) or event tickets (*n* = 54). The post with the most engagement (as defined by the number of likes) was a competition for entry to the popular music festival Rhythm & Vines (Figure 4). This post received 483 likes and over 4000 entries (by tagging a friend in the comments). Additional product prizes included e-cigarette devices and refills, branded merchandise (e.g., bucket hats, festival bags, drink bottles), and food vouchers, such as a post-festival ‘Hangover Brunch’.

### 3.5. Lifestyle Marketing

**Figure 5:**
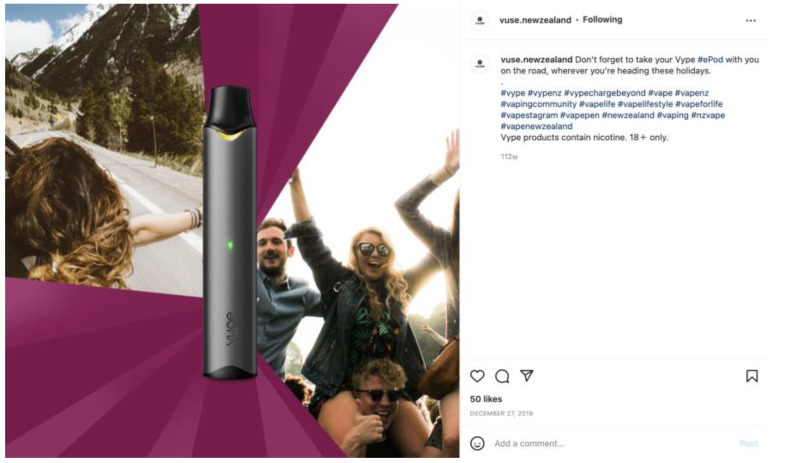
Example of an Instagram posts promoting lifestyle marketing.

**Figure 6:**
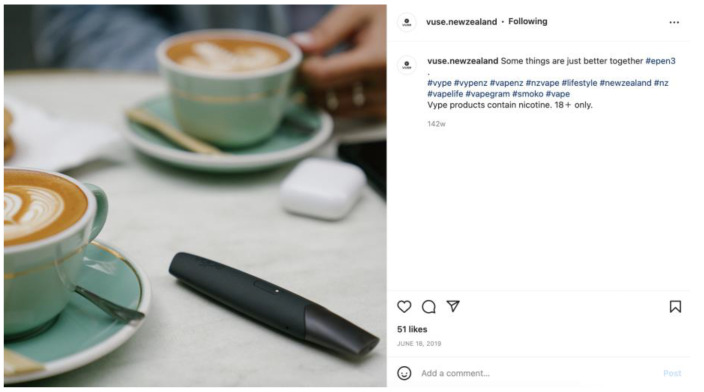
Example of an Instagram post positioning e-cigarettes with everyday items.

We also evaluated the use of lifestyle marketing, defined as marketing that presents positive associations or images of a way of life that involves “glamour, recreation, excitement, vitality, risk or daring” [31]. Of the 214 posts with lifestyle marketing, the majority featured people or models (*n =* 188) (Figure 5), while a smaller number of posts (*n =* 25) linked products with personal technology items such as smartphones, laptops, and café culture, positioning these products as part of everyday life (Figure 6).

### 3.6. Influencers

**Figure 7:**
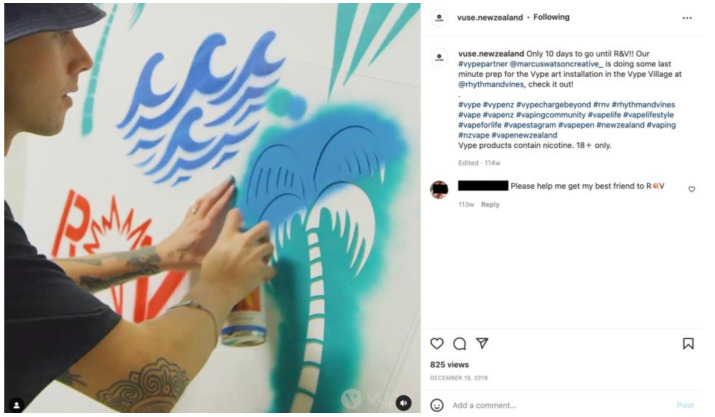
Example of an Instagram post with a social media influencer.

Influencers are defined in our study as an individual that is tagged using their Instagram handle with more than 500 followers. We identified 23 influencers in 47 posts. Influencers included DJs, models, sports people, television celebrities, artists, and designers (Figure 7). The number of followers ranged from 580 to 446,000 (mean 54,626, median 7798). Three influencers had a small following of between 500 and 1000. One influencer, aged 18 years at the time of this study (according to the age provided on their Instagram profile), was featured by two retailers in the sample.

### 3.7. Instagram Stories (24 h Posts)

**Figure 8:**
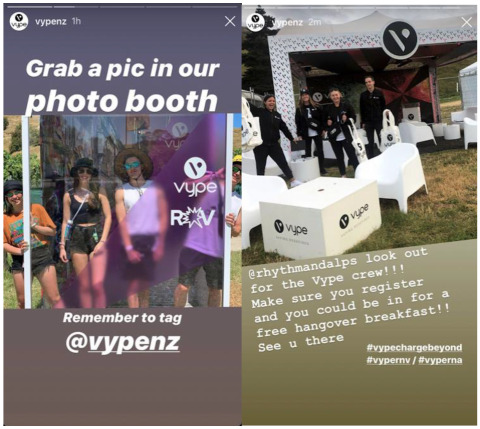
Example of Instagram Stories.

Instagram content uploaded using the Stories feature is not visible beyond a 24 h period (unless otherwise pinned by the user). Almost all Stories in the sample (*n* = 53/62) were related to festivals, for example, buying or sampling products at sponsored tents or concert crowds (Figure 8).

## 4. Discussion

Although the New Zealand e-cigarette industry has been found to position its products as a safer alternative to smoking [32], fewer than 10% of posts evaluated in this study referred to e-cigarettes as smoking replacements. Less than a third (28.1%) of posts included a statement about the product containing nicotine. Addiction warnings were rare, usually appearing in small font in post descriptions rather than directly on the main image. These findings are consistent with other studies, which show e-cigarette brands often lack health-related warnings and trivialise addiction on social media [13,14]. In this study, the retailers emphasised representations of style and individuality, such as limited edition designs and vivid colours. As Alpert et al. (2021) note, such features may appeal to a younger audience [14].

Instagram posts frequently depicted a lifestyle associated with youth culture, characterised by vitality, excitement, and glamour [31], situating products with youth-oriented activities, attitudes, and role models [12]. For decades, lifestyle marketing has been used in tobacco marketing [12] and, more recently, in the marketing of e-cigarettes [22,33]. The findings of this study suggest that New Zealand e-cigarette retailers may also use this strategy on Instagram to enhance social acceptance and expectancies. E-cigarette devices were placed alongside everyday items such as headphones and laptops and in cafe settings, normalising a highly addictive substance.

Almost a quarter of all posts in the sample featured music concerts and festivals, which were effective for engaging large groups of people with e-cigarette brands. For instance, in one post promoting a *Rhythm & Vines* ticket giveaway, more than 4000 people participated (by following the account and tagging friends to enter). The festival describes its audience as a “young, dynamic, disposable income kind of crowd” aged 18 to 25 years [34,35]. Whilst marketing to young adults, arguably ‘legal consumers’, may be viewed as acceptable, it is important to note that younger adolescents are particularly influenced by the lifestyle, consumption, and risk behaviours of older peers [36]. Twenty-four other music festivals and concerts were also promoted, including at least one with a minimum age of only 16 [37]. According to tobacco industry documents, music events have long been part of strategies to target young people described as “the key to future growth” [38]. Tobacco industry documents further state that these strategies facilitate “matching target groups’ lifestyle, attitudes and behaviour” [38].

Influencer or brand ambassador marketing is common on Instagram. As opposed to traditional advertising, influencers use endorsements, often interwoven with narratives, to promote products [39] for a fee or in exchange for products [29,39]. The current analysis of e-cigarette retailers’ Instagram posts in New Zealand is consistent with international studies that have documented the involvement of social media influencers in promoting e-cigarettes [7,40]. Influencers in this study included young artists, fashion designers, DJs, and chefs. Retailers may partner with influencers with large followings and youth appeal to expand their audience and connect e-cigarettes to youth culture. For example, one 18-year-old with over 40,000 Instagram followers who promoted products for two retailers in the sample, also shared the content on their personal Instagram account. Additionally, until 2020, Instagram influencers in New Zealand were not required to disclose paid content [41], making brand recommendations seem more authentic at the time of this study. However, the lack of official disclosure makes it impossible to determine how or if influencers were compensated. In December 2019, Instagram introduced policies prohibiting paid influencer content concerning e-cigarette products [42]. However, enforcement of these policies is inadequate, and violations are common [42].

Instagram Stories overtook Instagram posts as the most common Instagram format in 2019 [43]. The dynamic nature of Stories heightens user engagement, and this format is especially popular among young people [44]. Stories are based on ‘sharing a moment’, and this format enables users to add music and stickers to curate personalised and appealing content. The ephemeral nature of Stories may entice users to use the platform more regularly to ensure content is viewed. The majority of Instagram Stories in this study (53 of 62) depict festival-related content. For example, individuals sampling, displaying or promoting e-cigarette products and merchandise in branded tents and lounges. The use of Instagram Stories to emphasise festival content provides further evidence that young people are actively targeted by the industry using formats and content synonymous with youth culture.

The design and technology of social media platforms develops rapidly. Instagram recently introduced the video format Reels, which offers enhanced audio-video editing features. Reels are similar to the social media platform TikTok, which is popular among younger audiences. Future research should include the Reel format and any emerging social media trends, including exclusive paid subscriber content on Instagram [45]. The preferences for e-cigarette devices also change quickly. Disposable e-cigarettes are increasingly popular among young people as low-cost, high-nicotine products in many flavours and colours [46]. At the time of this study, we did not identify any disposable devices promoted on Instagram. However, we note that Vuse New Zealand introduced a range of disposable products on Instagram in October 2022 [47] which may indicate a shift from pod devices to disposable e-cigarettes.

A limitation of this study is that some posts may not have been captured, particularly the Stories withdrawn after 24 h. However, continuous monitoring during the study period sought to minimise this. Except for Stories, Instagram posts were gathered retrospectively, meaning companies could have removed some content. Second, the majority of coding was conducted by the first author, which may be viewed as a limitation. However, inter-coder reliability was high, and much of the data collected was objective, for example, the presence of an addiction warning or a ticket giveaway. Therefore, we are confident in our results.

The purpose of this study was to examine the social media marketing activities of e-cigarette retailers in New Zealand prior to the introduction of regulations. By examining marketing and promotion over this pre-regulation period, we are able to learn more about industry motivations and target audiences without the restrictions of tobacco control policies. Future research could evaluate the social media promotions made by e-cigarette retailers after the enactment of regulations, including on new and emerging platforms and post formats. The introduced regulations prohibit sponsorship and competitions, which may have curbed some youth-oriented marketing. However, there is evidence of industry adaption to circumvent these regulations on digital platforms [5]. Moreover, since social media is a global medium, companies can use global accounts to bypass country-specific regulations [48]. For example, the ‘worldwide’ account for Vuse promotes sponsored Formula One Racing and festivals, which is prohibited in New Zealand but visible to New Zealand Instagram users. It is unclear whether companies voluntarily age-gated their accounts during the study period. Further research is needed to determine whether the regulations designed to protect young people from e-cigarette marketing have been effective.

## 5. Conclusions

This study examined the marketing techniques and health claims presented by New Zealand-based e-cigarette retailers on social media. The findings suggest that e-cigarette companies deliberately target young people when unconstrained by regulation. Retailers utilised strategies similar to tobacco industry advertising tactics to engage with a young audience, including festival sponsorship and influencers popular with young people [49]. E-cigarette retailers claim to promote harm-reduction tools to smokers. Yet, this study finds few references to harm reduction or safer alternatives to smoking in any content, casting further doubt on industry motivations. Our findings suggest that the combination of weak and delayed government action and the openly youth-targeted marketing campaigns may have contributed to the high rate of e-cigarette use among young New Zealanders. Strong regulatory action and enforcement are needed to ensure retailers cannot utilise social media to target young people. Future research could evaluate the social media promotions made by e-cigarette retailers after the enactment of regulations, including on new and emerging platforms and post formats.

## Figures and Tables

**Table 1:** New Zealand e-cigarette retailer Instagram followers, posts, and Stories.

New Zealand Retailers	Followers (2019)	Stories *n =* 62	Posts *n* = 451 (%)	Average Likes per Image Post	Average Views per Video Post
Vapo	5274	47	113 (25.1%)	66.6	567
Vype	2869	8	129 (28.6%)	45.2	375
NZVapor	1238	0	122 (27.1%)	25.2	240
Cosmic	2540	4	65 (14.4%)	22.3	249
Shosha	2983	3	22 (4.9%)	37.6	675

**Table 2:** Characteristics of Instagram posts of New Zealand e-cigarette retailers.

Instagram Posts and Stories Characteristics	Details	Posts *n =* 451	Stories *n =* 62	Total *n* = 513	%
Health risks and benefits ***n*** = 233 (45.8%)	Smoking alternatives	48	2	50	9.7%
	Contains Nicotine	144	0	144	28.1%
	Addiction warnings	39	0	39	7.6%
Device type ***n*** = 211 (41.1%)	Pod	139	18	157	30.6%
	Modifiable	51	3	54	10.5%
	Disposable	0	0	0	0.0%
Product features ***n*** = 151 (29.7%)	Style	65	0	65	12.7%
	Ease of use	58	0	58	11.3%
	Quality	23	0	23	4.5%
	Environmentally friendly	5	0	5	1.0%
Featured events ***n*** = 117 (23.1%)	Music festivals/gigs	45	53	98	19.1%
	Motorsport	6	0	6	1.2%
	Expos	9	0	9	1.8%
	Other	4	0	4	0.8%
Giveaways and competitions ***n*** = 100 (19.7%)	Events	39	5	54	10.5%
	Products	41	5	46	9.0%
Lifestyle marketing ***n*** = 213 (41.5%)	Influencers (tagged)	39	8	47	9.2%
	People and models	151	37	188	36.6%
	Everyday items	25	1	25	4.9%
Price promotions ***n*** = 32 (6.3%)	Discounts	26	0	26	5.1%
	Deals	0	0	6	1.2%

## Data Availability

Not applicable.

## Supplementary Materials

The following supporting information can be downloaded at: https://www.mdpi.com/article/10.3390/ijerph20031897/s1, Table S1: Instagram Post and Story Characteristics: Definitions and Examples.

## References

[1] Peeters S., Gilmore A.B. (2015). Understanding the Emergence of the Tobacco Industry’s Use of the Term Tobacco Harm Reduction in Order to Inform Public Health Policy. Tob. Control.

[2] Hartmann-Boyce J., Lindson N., Butler A.R., McRobbie H., Bullen C., Begh R., Theodoulou A., Notley C., Rigotti N.A., Turner T. (2022). Electronic Cigarettes for Smoking Cessation. Cochrane Database Syst. Rev..

[3] Ministry of Health (2022). New Zealand Health Survey: Vaping/e-Cigarettes—Used at Least One a Day.

[4] World Health Organization (2021). WHO Report on the Global Tobacco Epidemic, 2021: Addressing New and Emerging Products.

[5] Cochran C., Robertson L., Hoek J. (2021). Online Marketing Activity Following New Zealand’s Vaping Legislation. Tob. Control.

[6] Boston A., Robertson L., Hoek J. (2022). Specialist Vape Store Developments during the Implementation of New Zealand’s Smokefree Environments and Regulated Products (Vaping) Amendment Act 2020. Tob. Control.

[7] O’Brien E.K., Hoffman L., Navarro M.A., Ganz O. (2020). Social Media Use by Leading US E-Cigarette, Cigarette, Smokeless Tobacco, Cigar and Hookah Brands. Tob. Control.

[8] Pokhrel P., Fagan P., Herzog T.A., Laestadius L., Buente W., Kawamoto C.T., Lee H.-R., Unger J.B. (2018). Social Media E-Cigarette Exposure and e-Cigarette Expectancies and Use among Young Adults. Addict. Behav..

[9] Kong G., LaVallee H., Rams A., Ramamurthi D., Krishnan-Sarin S. (2019). Promotion of Vape Tricks on YouTube: Content Analysis. J. Med. Internet Res..

[10] Kim M., Popova L., Halpern-Felsher B., Ling P.M. (2019). Effects of E-Cigarette Advertisements on Adolescents’ Perceptions of Cigarettes. Health Commun..

[11] Jackler R.K., Chau C., Getachew B.D., Whitcomb M.M., Lee-Heidenreich J., Bhatt A.M., Kim-O’Sullivan S.H.S., Hoffman Z.A., Jackler L.M., Ramamurthi D. JUUL Advertising Over Its First Three Years on the Market 48. https://tobacco-img.stanford.edu/wp-content/uploads/2021/07/21231836/JUUL_Marketing_Stanford.pdf.

[12] Ling P.M., Glantz S.A. (2002). Why and How the Tobacco Industry Sells Cigarettes to Young Adults: Evidence From Industry Documents. Am. J. Public Health.

[13] Czaplicki L., Kostygina G., Kim Y., Perks S.N., Szczypka G., Emery S.L., Vallone D., Hair E.C. (2019). Characterising JUUL-Related Posts on Instagram. Tob. Control.

[14] Alpert J.M., Chen H., Riddell H., Chung Y.J., Mu Y.A. (2021). Vaping and Instagram: A Content Analysis of e-Cigarette Posts Using the Content Appealing to Youth (CAY) Index. Subst. Use Misuse.

[15] Most Used Social Media 2021. https://www.statista.com/statistics/272014/global-social-networks-ranked-by-number-of-users/.

[16] (2019). NapoleonCat.Stats. “Instagram Users in New Zealand”. NapoleonCat. https://napoleoncat.com/stats/instagram-users-in-new_zealand/2019/02/#:~:text=There%20were%201%20711%20000,user%20group%20(520%20000).

[17] Li Y., Xie Y. (2020). Is a Picture Worth a Thousand Words? An Empirical Study of Image Content and Social Media Engagement. J. Mark. Res..

[18] Alpert J.M., Chen H., Adams K.-A. (2019). E-Cigarettes and Social Media: Attitudes and Perceptions of Young Adults to Social Media Messages. Addict. Res. Theory.

[19] Barry A.E., Padon A.A., Whiteman S.D., Hicks K.K., Carreon A.K., Crowell J.R., Willingham K.L., Merianos A.L. (2018). Alcohol Advertising on Social Media: Examining the Content of Popular Alcohol Brands on Instagram. Subst. Use Misuse.

[20] Richardson A., Ganz O., Stalgaitis C., Abrams D., Vallone D. (2014). Noncombustible Tobacco Product Advertising: How Companies Are Selling the New Face of Tobacco. Nicotine Tob. Res..

[21] Hoek J., Freeman B. (2019). BAT(NZ) Draws on Cigarette Marketing Tactics to Launch Vype in New Zealand. Tob. Control.

[22] Stevens E.M., Johnson A.L., Leshner G., Sun F., Kim S., Leavens E.L.S., Tackett A.P., Hébert E.T., Wagener T.L. (2020). People in E-Cigarette Ads Attract More Attention: An Eye-Tracking Study. Tob. Regul. Sci..

[23] Wagner N. (2017). Nicotine E-Cigarettes to Become Legal.

[24] New Zealand District Court (2018). Case 4478 Phillip Morris New Zealand Ltd v Ministry of Health.

[25] ASA—Advertising Standards Authority. https://www.asa.co.nz/.

[26] Gurram N., Thomson G., Wilson N., Hoek J. (2019). Electronic Cigarette Online Marketing by New Zealand Vendors. New Zealand Med. J..

[27] Hardie L., McCool J., Freeman B. (2021). Online Retail Promotion of E-Cigarettes in New Zealand: A Content Analysis of e-Cigarette Retailers in a Regulatory Void. Health Promot. J. Aust..

[28] Kite J., Foley B.C., Grunseit A.C., Freeman B. (2016). Please Like Me: Facebook and Public Health Communication. PLoS ONE.

[29] Vassey J., Valente T., Barker J., Stanton C., Li D., Laestadius L., Cruz T.B., Unger J.B. (2022). E-Cigarette Brands and Social Media Influencers on Instagram: A Social Network Analysis. Tob. Control.

[30] Wies S., Bleier A., Edeling A. (2022). Finding Goldilocks Influencers: How Follower Count Drives Social Media Engagement. J. Mark..

[31] Government of Canada (2020). Consolidated Federal Laws of Canada, Tobacco and Vaping Products Act.

[32] Hardie L., McCool J., Freeman B. (2022). Use of Supporting Evidence by Health and Industry Organisations in the Consultation on E-Cigarette Regulations in New Zealand. PLoS ONE.

[33] Czaplicki L., Tulsiani S., Kostygina G., Feng M., Kim Y., Perks S.N., Emery S., Schillo B. (2020). #toolittletoolate: JUUL-Related Content on Instagram before and after Self-Regulatory Action. PLoS ONE.

[34] McConnell G. The Secret to 16 Years of Rhythm and Vines: It’s All about the Vibe. *Stuff*
**2019**. https://www.stuff.co.nz/entertainment/music/109707357/the-secret-to-16-years-of-rhythm-and-vines-its-all-about-the-vibe.

[35] Hoksbergen E., Insch A. (2016). Facebook as a Platform for Co-Creating Music Festival Experiences: The Case of New Zealand’s Rhythm and Vines New Year’s Eve Festival. Int. J. Event Festiv. Manag..

[36] Knoll L.J., Magis-Weinberg L., Speekenbrink M., Blakemore S.-J. (2015). Social Influence on Risk Perception During Adolescence. Psychol. Sci..

[37] FAQ—Soundsplash. https://soundsplash.co.nz/faqs/.

[38] Benson & Hedges Music—Positioning Paper—Truth Tobacco Industry Documents. https://www.industrydocuments.ucsf.edu/tobacco/docs/#id=yswg0192.

[39] De Veirman M., Cauberghe V., Hudders L. (2017). Marketing through Instagram Influencers: The Impact of Number of Followers and Product Divergence on Brand Attitude. Int. J. Advert..

[40] Klein E.G., Czaplicki L., Berman M., Emery S., Schillo B. (2020). Visual Attention to the Use of #ad versus #sponsored on E-Cigarette Influencer Posts on Social Media: A Randomized Experiment. J. Health Commun..

[41] ASA—Advertising Standards Authority Guidance Note on Identification of Advertisements. https://www.asa.co.nz/codes/code-guidance-notes/guidance-note-identification-advertisements/.

[42] Kong G., Laestadius L., Vassey J., Majmundar A., Stroup A.M., Meissner H.I., Ben Taleb Z., Cruz T.B., Emery S.L., Romer D. (2022). Tobacco Promotion Restriction Policies on Social Media. Tob. Control.

[43] Bainotti L., Caliandro A., Gandini A. (2020). From Archive Cultures to Ephemeral Content, and Back: Studying Instagram Stories with Digital Methods. New Media Soc..

[44] Belanche D., Cenjor I., Pérez-Rueda A. (2019). Instagram Stories versus Facebook Wall: An Advertising Effectiveness Analysis. Span. J. Mark.-ESIC.

[45] Instagram Subscriptions: Learn How to Use Instagram’s Paid Subscriptions to Earn Monthly Income. https://creators.instagram.com/earn-money/subscriptions?locale=en_GB.

[46] Watts C., Egger S., Dessaix A., Brooks A., Jenkinson E., Grogan P., Freeman B. (2022). Vaping Product Access and Use among 14–17-Year-Olds in New South Wales: A Cross-Sectional Study. Aust. New Zealand J. Public Health.

[47] Vusionry/Vuse NZ (@vuse.Newzealand) Instagram Photos and Videos. https://www.instagram.com/vuse.newzealand/.

[48] Freeman B., Watts C., Astuti P.A.S. (2022). Global Tobacco Advertising, Promotion and Sponsorship Regulation: What’s Old, What’s New and Where to next?. Tob. Control.

[49] Chu K.-H., Allem J.-P., Cruz T.B., Unger J.B. (2017). Vaping on Instagram: Cloud Chasing, Hand Checks and Product Placement. Tob. Control.

