# Supplementary material for: E-Cigarette Retailers’ Use of Instagram in New Zealand: A Content Analysis
Source: Int J Environ Res Public Health. 2023 Jan 19;20(3):1897. doi: 10.3390/ijerph20031897 (PMC9914635; doi:10.3390/ijerph20031897)
Supplement: Supplementary file 1 [file ijerph-20-01897-s001.zip › ijerph-2095354-supplementary.pdf]

Table S1. Instagram Post and Story Characteristics: Definitions and Examples.

<table>
<tr><th>Details</th><th>Instagram Post and Story Characteristics: Definitions and Examples</th><th>Total
n = 513</th><th>%</th></tr>
<tr><td colspan="4">**Health Risks and Benefits**</td></tr>
<tr><td>**Smoking cessation**</td><td>Post contains references to smoking, quitting, switching from combustible cigarettes and/or “make the switch” or similar.
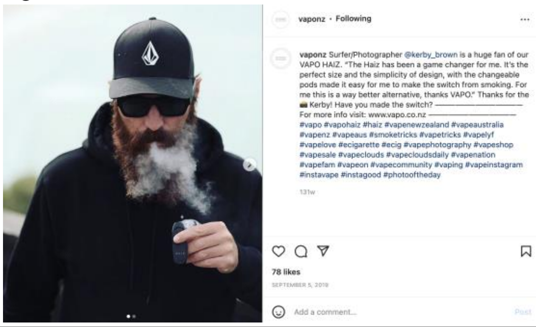</td><td>50</td><td>9.7%</td></tr>
<tr><td>**Contains Nicotine**</td><td>Post contains statements that product contains nicotine.
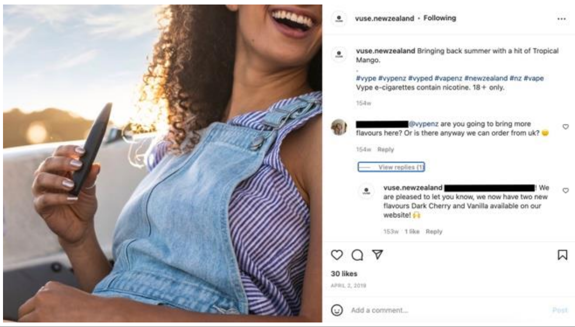</td><td>144</td><td>28.1%</td></tr>
<tr><td>**Addiction warnings**</td><td>Post contains warnings about nicotine addiction and/or "Nicotine is an addictive substance" or similar.
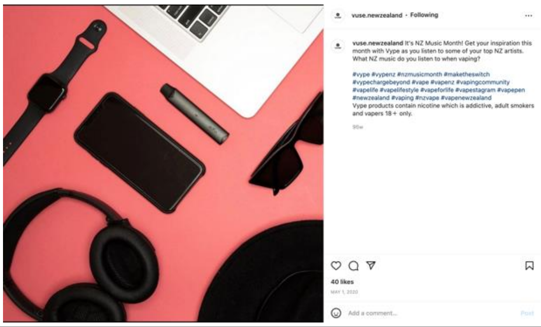</td><td>39</td><td>7.6%</td></tr>
<tr><td colspan="4">**Featured Events**</td></tr>
<tr><td>**Music festivals/gigs**</td><td>Post contains references to organised music concerts or series of concerts (festival).</td><td>98</td><td>19.1%</td></tr>
</table>

	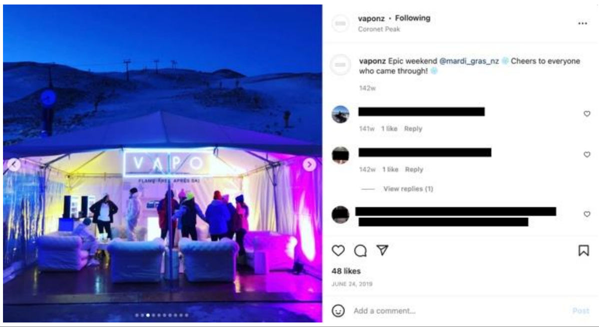		
**Motorsport**	Post contains references to a sports event involving racing cars or other motor vehicle events.<br>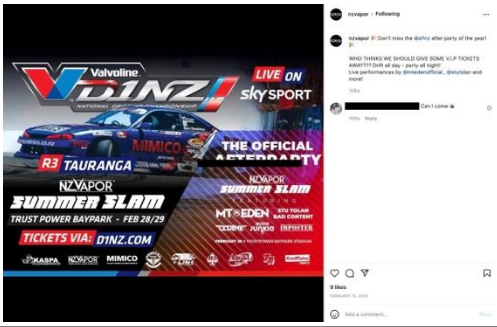	6	1.2%
**Expos**	Post contains references to an exhibition event tradeshow.<br>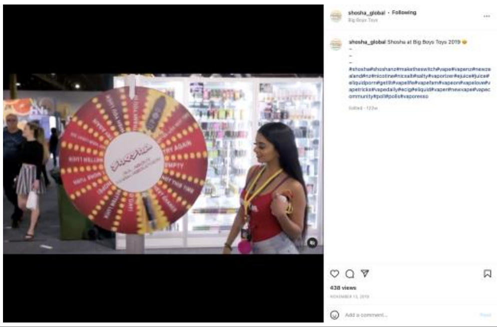	9	1.8%
**Other**	Post contains references to other events including sporting events e.g., boxing matches, awards ceremonies or other.<br>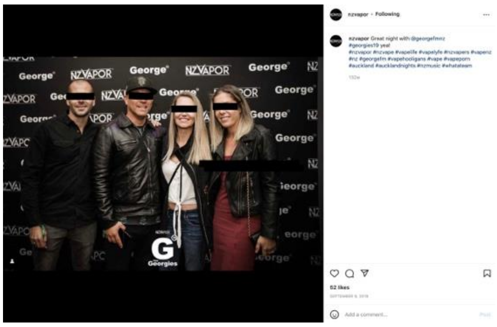	4	0.8%
	**Giveaways and competitions**		
**Events**	Post contains a competition or giveaway where the prize is event entry.	54	10.5%

<table>
<tr><th></th><th></th><th></th><th></th></tr>
<tr><td></td><td>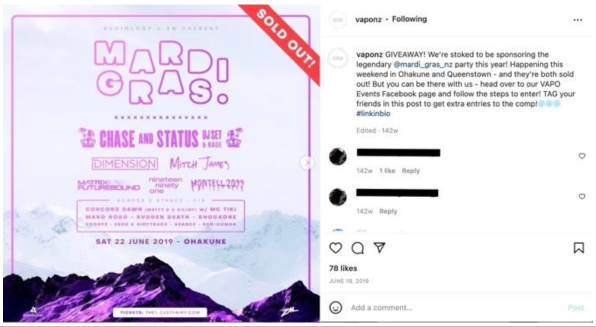
</td><td></td><td></td></tr>
<tr><td>Products</td><td>Post contains a competition or giveaway where prizes are products including e-cigarette devices, e-liquid refills, food vouchers, vouchers and/or other products.
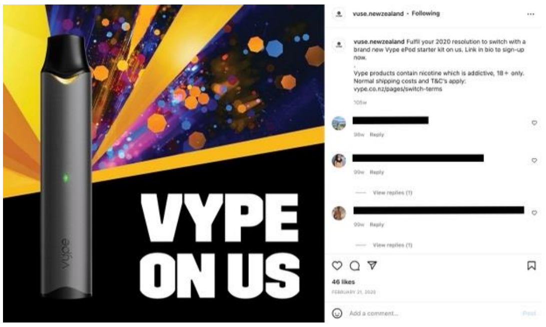
</td><td>46</td><td>9.0%</td></tr>
<tr><td colspan="4">Lifestyle marketing</td></tr>
<tr><td>Influencers (tagged)</td><td>Post contains individuals with more than 500 followers AND are tagged in the post using their Instagram handle e.g., "@XXXX"
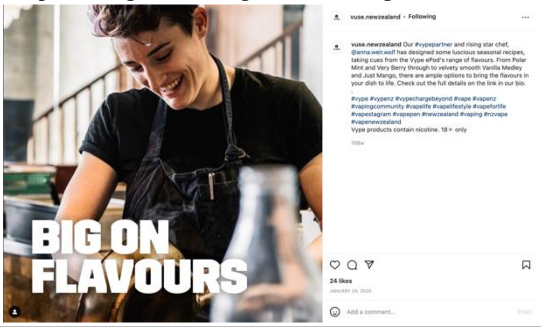
</td><td>47</td><td>9.2%</td></tr>
<tr><td>Models/People</td><td>Post contains individuals who are paid models, or members of the public who feature in posts AND are not tagged using their Instagram handle
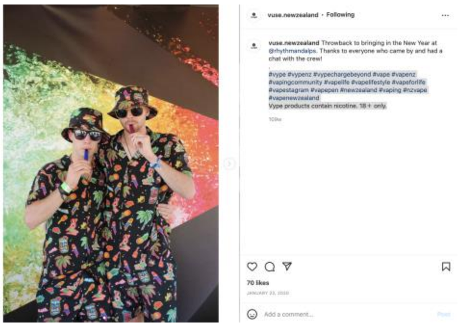
</td><td>188</td><td>36.6%</td></tr>
<tr><td>Everyday items</td><td>Post contains personal and everyday items including laptops, headphones, smartphones and/or cameras.</td><td>25</td><td>4.9%</td></tr>
</table>

	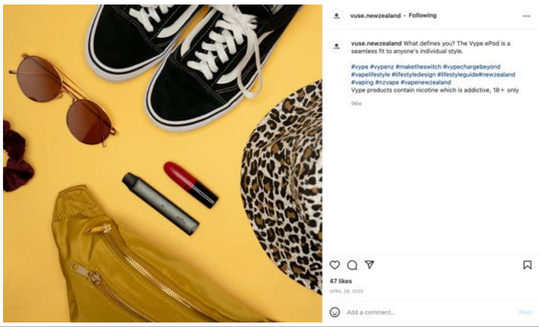		
**Devices**			
**Pod**	Post contains a compact e-cigarette device that uses a pod chamber to hold e-liquid.<br>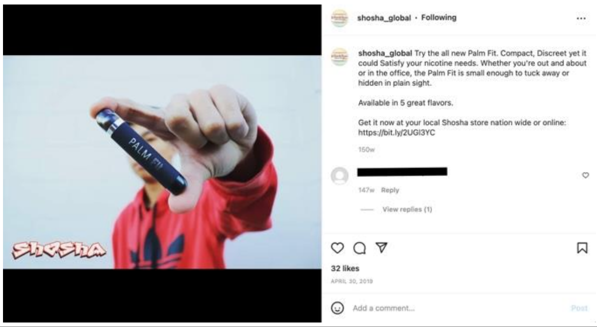	157	30.6%
**Modifiable**	Post contains a modifiable "mod" device with refillable chambers to hold e-liquid.<br>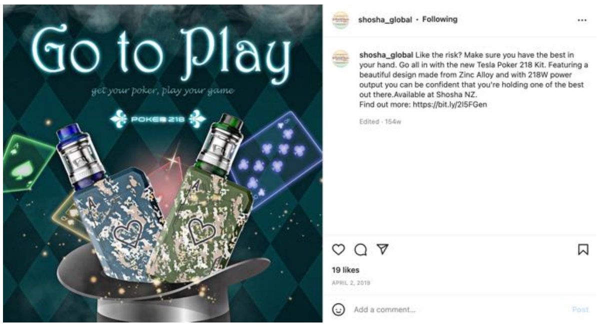	54	10.5%
**Disposable**	Post contains a disposable single use device<br>-	0	0.0%
**Product Features**			
**Style**	Post contains statements that refer to aspects of style such as colour, individuality and/or discrete size.<br>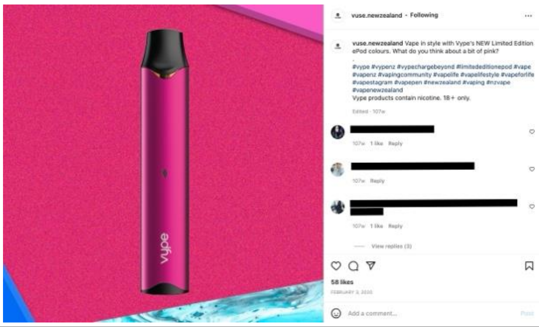	65	12.7%
**Ease of use**	Post contains statements that refer to design features that make the product	58	11.3%

<table>
<tr><td></td><td>simple to use including referencing to being easy to use and/or for beginners
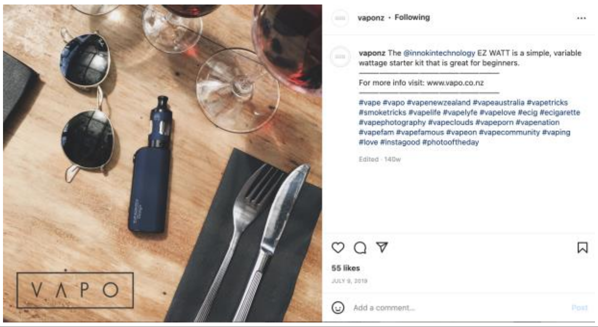
</td><td></td><td></td></tr>
<tr><td>Quality</td><td>Post contains aspects of quality such as scientific standards.
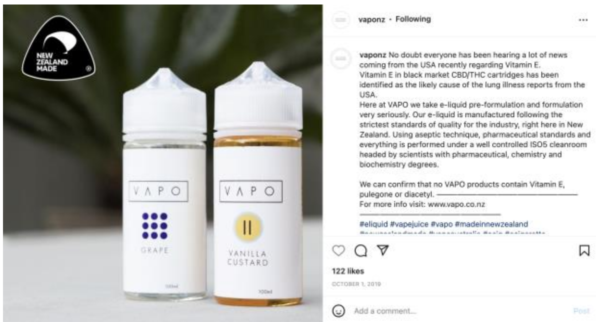
</td><td>23</td><td>4.5%</td></tr>
<tr><td>Environmentally friendly</td><td>Post contains aspects of positive environmental impacts including recycling, biodegradable materials, sustainability and/or palm oil free.
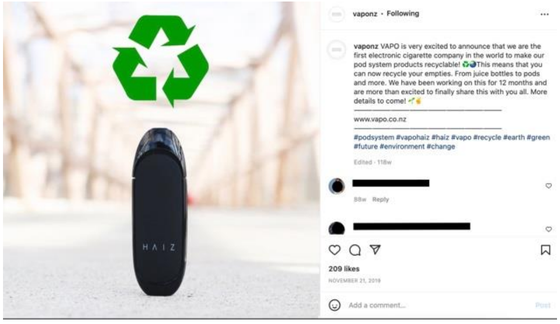
</td><td>5</td><td>1.0%</td></tr>
<tr><td colspan="4">Price Promotions</td></tr>
<tr><td>Discounts</td><td>Post contains references to price discounts.
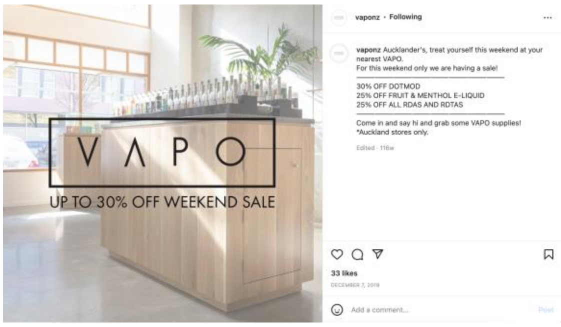
</td><td>26</td><td>5.1%</td></tr>
<tr><td>Deals</td><td>Post contains references to pricing deals including bundles, gifts with purchase.</td><td>6</td><td>1.2%</td></tr>
</table>

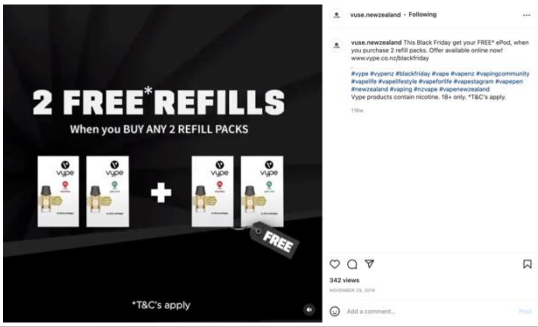
2 FREE* REFILLS
When you BUY ANY 2 REFILL PACKS
FREE
*T&C's apply
vuse.newzealand • Following
vuse.newzealand This Black Friday get your FREE* ePod, when you purchase 2 refill packs. Offer available online now! www.vype.co.nz/blackfriday
#vype #vypenz #blackfriday #vape #vapenz #vapingcommunity #vapelife #vapelifestyle #vapeforlife #vapestagram #vapepen #newzealand #vaping #nzvape #vapenewzealand
Vype products contain nicotine. 18+ only. *T&C's apply.
342 views
Add a comment...
